# Early Intervention With Boiogito to Suppress Knee Osteoarthritis Progression: An Experimental Approach Using a Medial Meniscus Instability Rat Model

**DOI:** 10.7759/cureus.77311

**Published:** 2025-01-12

**Authors:** Kanako Izukashi, Takayuki Okumo, Tokito Tatsuo, Itaru Kachi, Yuta Iida, Takumi Nishio, Hideshi Ikemoto, Naoki Adachi, Koji Kanzaki, Masataka Sunagawa

**Affiliations:** 1 Department of Physiology, Showa University Graduate School of Medicine, Tokyo, JPN; 2 Department of Orthopedic Surgery, Showa University Fujigaoka Hospital, Yokohama, JPN

**Keywords:** articular cartilage, boiogito, fangji huangqi tang, knee osteoarthritis (koa), medial meniscus, osteoclast, rat model, subchondral bone

## Abstract

Background

Knee osteoarthritis (KOA) is a prevalent and chronic condition characterized by swelling, pain, and limited range of motion of the knee due to degenerative changes in joint structures, leading to impairment in performing daily activities. Although conservative treatments, such as exercise therapy and nonsteroidal anti-inflammatory drugs are employed, there are few effective therapeutic options for preventing disease progression. During early KOA, there is osteoclast proliferation in the subchondral bone, disruption in cartilage homeostasis, elevation of matrix metalloproteinase-13 (MMP-13) levels, and reduction in tissue inhibitors of matrix metalloproteinase-1 (TIMP-1) levels. Boiogito (BOT), which is a traditional Japanese medicinal formula, attenuates KOA progression, however, its effects when administered after KOA progression remain unclear. This study aimed to assess the therapeutic efficacy of BOT in preventing KOA progression in a rat model by focusing on its effects on motor function, subchondral bone turnover, and cartilage degradation in relation to the timing of administration.

Methods

A rat KOA model was created by destabilizing the medial meniscus (DMM). Rats were divided into Sham, DMM, DMM + BOT (0w, BOT administered immediately post-surgery), and DMM + BOT (3w, BOT administered 3 weeks post-surgery) groups. BOT was included in the diet at 1% (w/w). Motor function was evaluated biweekly by a treadmill running test, while structural changes in the knee were assessed by measuring the medial meniscus extrusion ratio (MMER) using computed tomography (CT). Histological and immunohistochemical analyses were conducted to evaluate joint degeneration via the Osteoarthritis Research Society International (OARSI) score, osteoclast numbers in subchondral bone through tartrate-resistant acid phosphatase (TRAP) staining, and MMP-13/TIMP-1 ratios in articular cartilage.

Results

Treadmill testing revealed that the DMM + BOT (0w) had significantly higher running speeds compared with the DMM and DMM + BOT (3w) groups. In all groups that underwent DMM surgery, the MMER was not significantly different. Histological assessments showed that the DMM + BOT (0w) group had lower OARSI scores and reduced osteoclast numbers in the subchondral bone compared with the DMM group. Immunohistochemical analysis showed a significant reduction in MMP-13 expression and MMP-13/TIMP-1 ratios in the DMM + BOT (0w) group, whereas the DMM + BOT (3w) group showed limited efficacy compared with the early intervention.

Conclusion

Early administration of BOT attenuates KOA progression by preserving motor function, reducing subchondral bone turnover, and mitigating cartilage degradation. These findings highlight the importance of early intervention with BOT to achieve optimal therapeutic outcomes in KOA.

## Introduction

Knee osteoarthritis (KOA) is a chronic degenerative joint condition characterized by swelling, pain, and reduced range of motion that significantly impairs the performance of daily activities [1]. In 2020, approximately 595 million individuals worldwide (7.6% of the world population) were affected by osteoarthritis, which will increase by 74.9% by 2050 [2]. Treatment of KOA consists of conservative and surgical approaches. Conservative treatment includes patient education and exercise therapy as well as symptomatic management using nonsteroidal anti-inflammatory drugs (NSAIDs) and corticosteroids [3]. Meanwhile, surgical treatment is reserved for severe cases that are refractory to conservative treatment.

Iijima et al. [4,5] demonstrated that osteoclast proliferation in the subchondral bone is an important factor for disease progression in meniscus injury-induced KOA model rats. Additionally, upregulated cartilage resorption, as represented by increased MMP-13 expression and decreased TIMP-1 levels, highlights the disruption of cartilage homeostasis [6]. These pathological changes can be mimicked with surgical techniques such as destabilization of the medial meniscus (DMM) and anterior cruciate ligament (ACL) transection.

Effective therapies targeting the underlying pathology of KOA remain elusive. Boiogito (BOT), which is a traditional Japanese medicinal formula known as Fangji Huangqi Tang in Chinese medicine, attenuates KOA progression [7]. Kimura et al. [8] demonstrated that BOT suppresses osteoclast proliferation in the subchondral bone in KOA model rats. Although KOA onset and progression in rat models occurred within 2 weeks post-induction [9], studies have administered BOT immediately after model establishment, leaving its efficacy in later disease stages unexplored.

Therefore, this study aimed to assess the effects of BOT on KOA progression through histological and immunohistochemical evaluations in a rat model of KOA to provide insights into its potential as a therapeutic intervention for advanced KOA.

## Materials and methods

**Figure 1:**
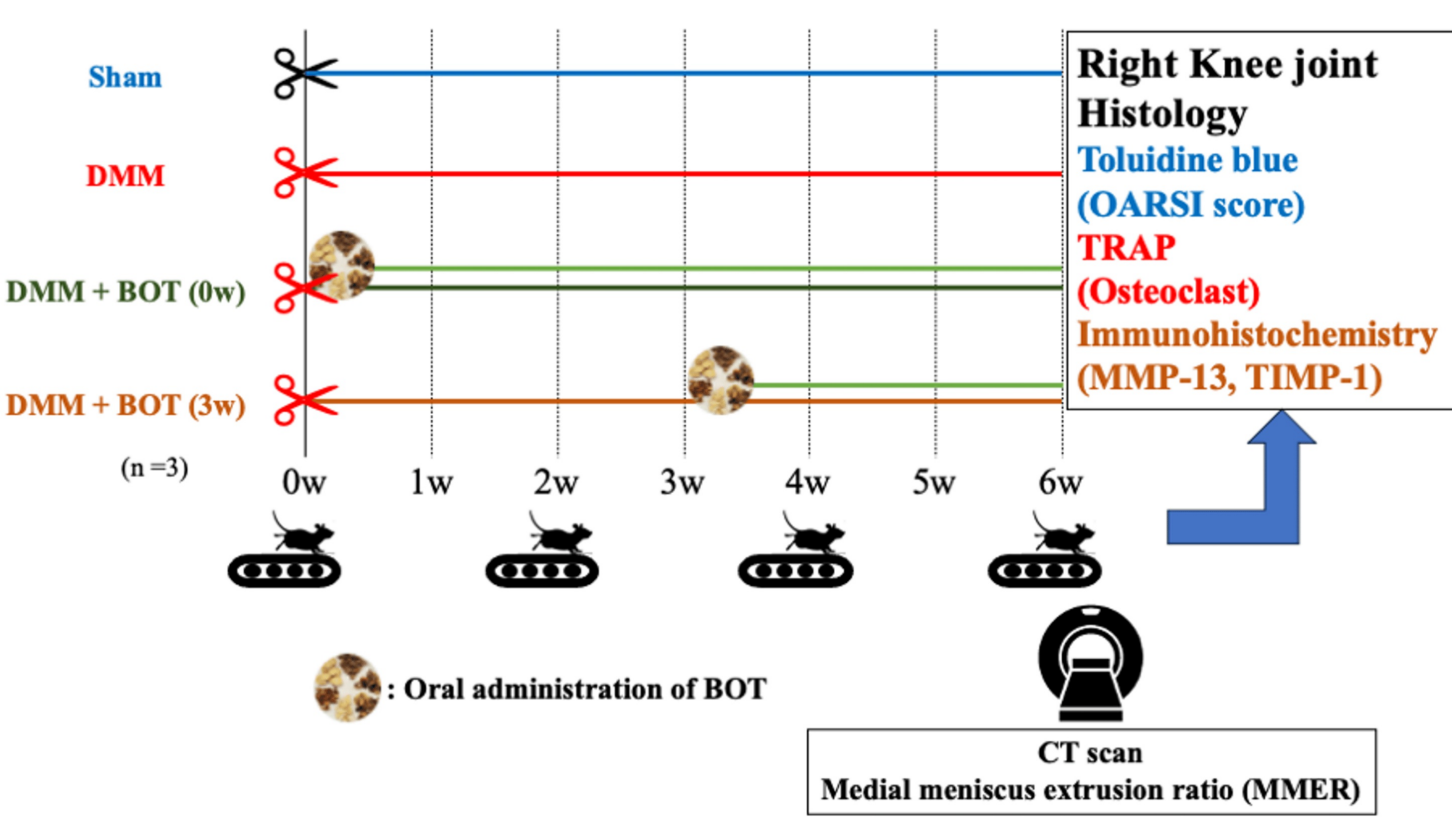
Experimental protocol Ten-week-old male Wistar rats underwent DMM surgery in the right knee joint. The animals were divided into the Sham, DMM, DMM + BOT (0w), and DMM + BOT (3w) groups. Throughout the 6-week experimental period, treadmill running tests were periodically performed. At 6 weeks, CT was conducted followed by histological and immunohistochemical staining of the right knee joint. BOT: boiogito, CT: computed tomography, DMM: destabilization of the medial meniscus, OARSI: Osteoarthritis Research Society International, MMP-13: matrix metalloproteinase-13, TIMP-1: tissue inhibitors of matrix metalloproteinase-1, TRAP: tartrate-resistant acid phosphatase Image Credit: Kanako Izukashi

The study protocol was approved by the Ethical Committee of the Showa University Animal Laboratory (approval no. #124053) (Fig. 1). In this study, 10-week-old male Wistar rats were used. The rats were housed in cages (three/cage) that were kept at 24°-26°C, with a humidity of 40%-60% and a 12-h light/dark cycle. DMM was performed on the right knee on 18 March 2024, and the rats were divided into four groups thereafter: DMM group, DMM + BOT (0w) group, DMM + BOT (3w) group, and Sham group, wherein only identification of the medial meniscus was performed without destabilization. BOT was mixed with powdered chow at 1% (w/w) using a medical-grade Kampo extract. To evaluate motor function, treadmill testing was conducted before DMM surgery and at 2-week intervals following surgery. After six weeks, on 29 April 2024, computed tomography (CT) scans were performed on the right knee, and the medial meniscus extrusion ratio (MMER) was measured. Following euthanasia, the right knee joints were harvested for histological and immunohistochemical assessments. KOA progression was evaluated using the Osteoarthritis Research Society International (OARSI) score via toluidine blue staining. Osteoclast numbers in the subchondral bone were measured via tartrate-resistant acid phosphatase (TRAP) staining, and the matrix metalloproteinase-13 (MMP-13)/tissue inhibitors of matrix metalloproteinase-1 (TIMP-1) ratio was obtained based on immunohistochemical staining for MMP-13 and TIMP-1. The four groups were then compared using statistical analysis.

The DMM group was considered the KOA-induced rat model due to its similarity to the pathogenesis of KOA in humans [10]. Following an earlier report [11], DMM surgery was performed under general anesthesia with isoflurane inhalation. A longitudinal incision was made on the right knee joint, and the medial meniscotibial ligament (MMTL) was exposed by dissecting the medial joint capsule. MMTL transection resulted in the loss of stability and hoop function of the medial meniscus. The medial capsule and skin were sutured using 6-0 Vicryl (Ethicon, Inc., Somerville, NJ, USA). 

The treadmill running test was conducted to evaluate motor function [12]. Following acclimatization, the rats started running at a gradually increasing speed, initially at 24 m/min for 1 min that was increased to 30 m/min for 1 min. The treadmill speed was then increased by 1 m/min at 20-s intervals until the rats could no longer run. The duration of running was obtained, and their endurance was assessed by measuring the maximum running speed. The rats preformed the treadmill running test before DMM surgery and at biweekly intervals following KOA induction.

Meniscus extrusion resulting from DMM surgery was assessed using CT [11]. The MMER was obtained from a coronal section after determining the axial and sagittal reference lines that showed the medial meniscus at its widest. The MMER was calculated as the proportion of the medial meniscus that protruded beyond the edge of the tibial plateau to its total transverse length; a higher value indicates greater extrusion. As DMM surgery destabilizes the medial meniscus, an increase in MMER is expected.

For histological analysis, the rats were anesthetized with intraperitoneal sodium pentobarbital (50 mg/kg; Somnopentyl, Kyoritsu Seiyaku, Tokyo, Japan) and perfused with phosphate-buffered saline (pH 7.4). The knee joint was then fixed using 4% paraformaldehyde. Tissue slices of the knee joint were prepared according to the OARSI guidelines [13]. Briefly, samples were fixed in 4% paraformaldehyde for 3 days and demineralized in ethylenediaminetetraacetic acid disodium (EDTA-2Na) over a 3-week period. Knee joints were then bisected at the midpoint of the tibial anteroposterior diameter approximately along with the medial collateral ligament, embedded in paraffin, and sectioned into 4-µm slices using a Retratome (REM-700, Yamato Kohki Industrial Co., Ltd., Saitama, Japan) at 200-µm intervals. The slices were mounted on glass slides, dehydrated, stained with toluidine blue, and examined under an Olympus BX53 microscope (Olympus, Tokyo, Japan). Joint degeneration was evaluated using the OARSI scoring system for rats, which assigns scores for cartilage degeneration (0-15 points), subchondral bone destruction (0-5 points), synovitis (0-4 points), and osteophyte formation (0-4 points), with the total score ranging from 0 to 28; low scores indicate lower joint damage. Additionally, subchondral bone turnover was evaluated in early-stage KOA by counting TRAP-positive osteoclasts, which were identified as multinucleated giant cells [14], using a TRAP staining kit (Fujifilm Wako Pure Chemical Corporation, Osaka, Japan).

Immunohistochemical analysis of MMP-13 and TIMP-1 was conducted on tibial articular surface sections [15]. Following three 3-minute washes with tris-buffered saline (TBS), endogenous peroxidase activity was inhibited using a 0.3% H_2_O_2_/methanol solution for 15 min. Sections were then blocked with 5% bovine serum albumin and 0.2% Triton X-100 (Fujifilm Wako Pure Chemical Corporation, Osaka, Japan) in Phosphate-buffered saline (PBS) for 1 h. Subsequently, sections were incubated overnight at 4°C with either a rabbit monoclonal anti-MMP-13 antibody (#69926, Cell Signaling Technology, Tokyo, Japan; dilution 1:200) or an anti-TIMP-1 antibody (#8946, Cell Signaling Technology, Tokyo, Japan; dilution 1:200). To visualize the immunoreaction, a streptavidin-biotin-peroxidase complex was applied at room temperature using the IHC Select® horseradish peroxidase/3,3′-diaminobenzidine (HRP/DAB) Test kit (Sigma-Aldrich, Germany). The SignalStain® DAB Substrate Kit (#8059, Cell Signaling Technology, Tokyo, Japan) was used for staining, while hematoxylin was used to counterstain cell nuclei. All sections were examined under an Olympus BX53 microscope (Olympus, Tokyo, Japan). Semiquantitative analysis [16] was conducted to assess the percentage of positive cells and staining intensity for MMP-13 and TIMP-1, with evaluations performed on an ordinal scale ranging from 0 to 8 points. The percentage of positive cells was graded on a scale of 0-5, with scores corresponding to the proportion of positively stained cells and/or matrix (0: no staining; 1: <5%; 2: 6%-24%; 3: 25%-49%; 4: 50%-75%; 5: >75%). Staining intensity was rated on a scale of 0-3 (0: no staining; 1: minimal; 2: moderate; 3: marked). The combined scores, ranging from 0 to 8, indicated the severity of cartilage degeneration. Analysis was conducted using randomly numbered sections under 200× magnification with light microscopy.

Data for each group are presented as mean ± standard error. Statistical analyses were performed using JMP Pro version 17.0 software (SAS Inc., Cary, NC, USA). Significance was assessed using the Tukey test and set at a P-value of 0.05.

## Results

**Figure 2:**
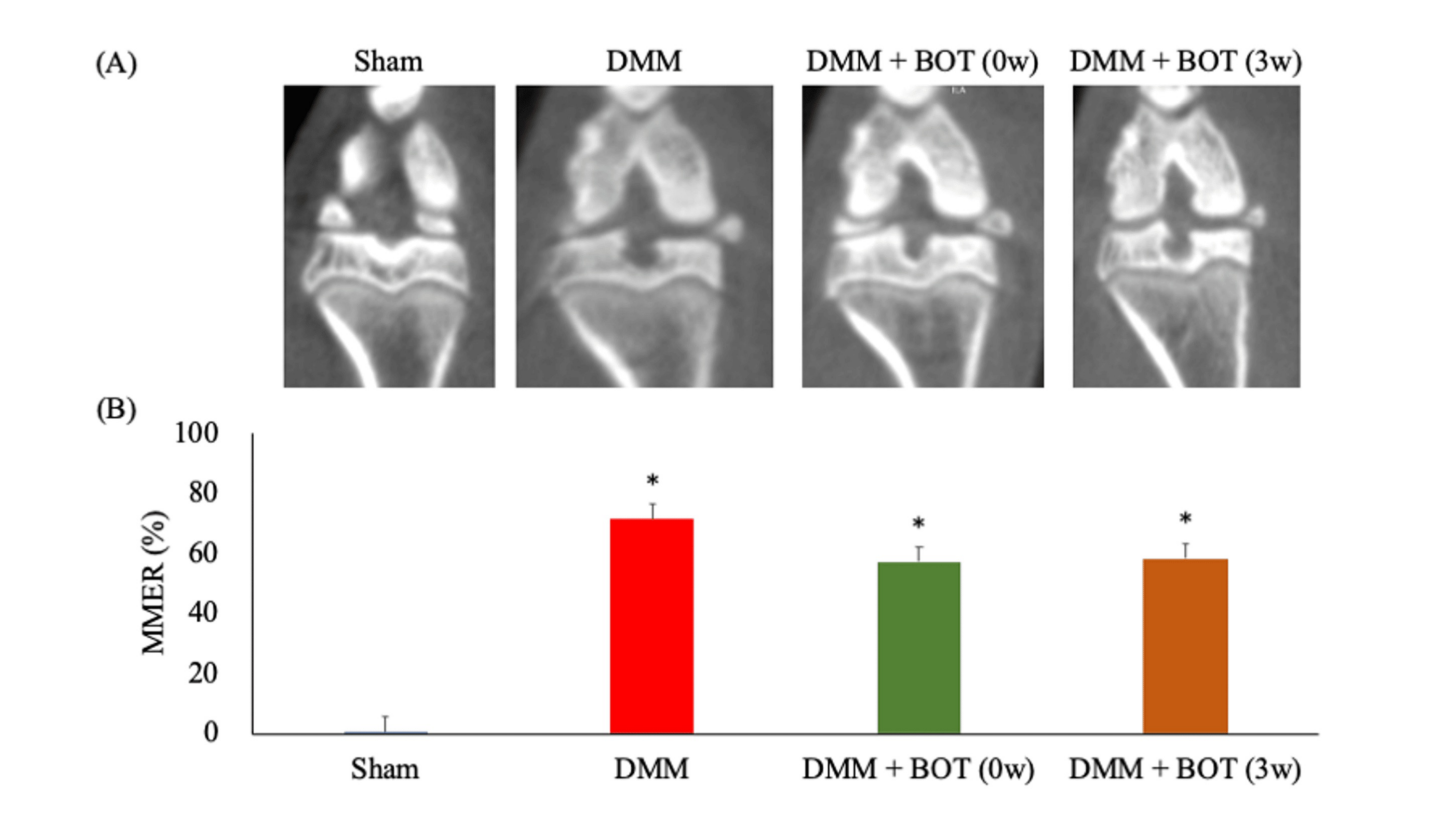
CT-based analysis of the meniscus extrusion ratio (A) Representative CT images at week 6 after DMM surgery. The images showing the widest appearance of the medial meniscus in the coronal plane were selected, and the width of the extruded meniscus from the medial edge of the tibia was measured relative to the width of the medial meniscus. (B) MMER. *P < 0.05 vs. the Sham group based on the Tukey test. BOT: boiogito, CT: computed tomography, DMM: destabilization of the medial meniscus, MMER: medial meniscus extrusion ratio.

Six weeks after DMM surgery, the MMER was 0.5% ± 0.5% in the Sham group, 71.4% ± 8.7% in the DMM group, 57.2% ± 3.7% in the DMM + BOT (0w) group, and 58.1% ± 13.8% in the DMM + BOT (3w) group (Fig. 2A). These results indicate that MMER increased in all groups that underwent DMM surgery (Fig. 2B).

**Figure 3:**
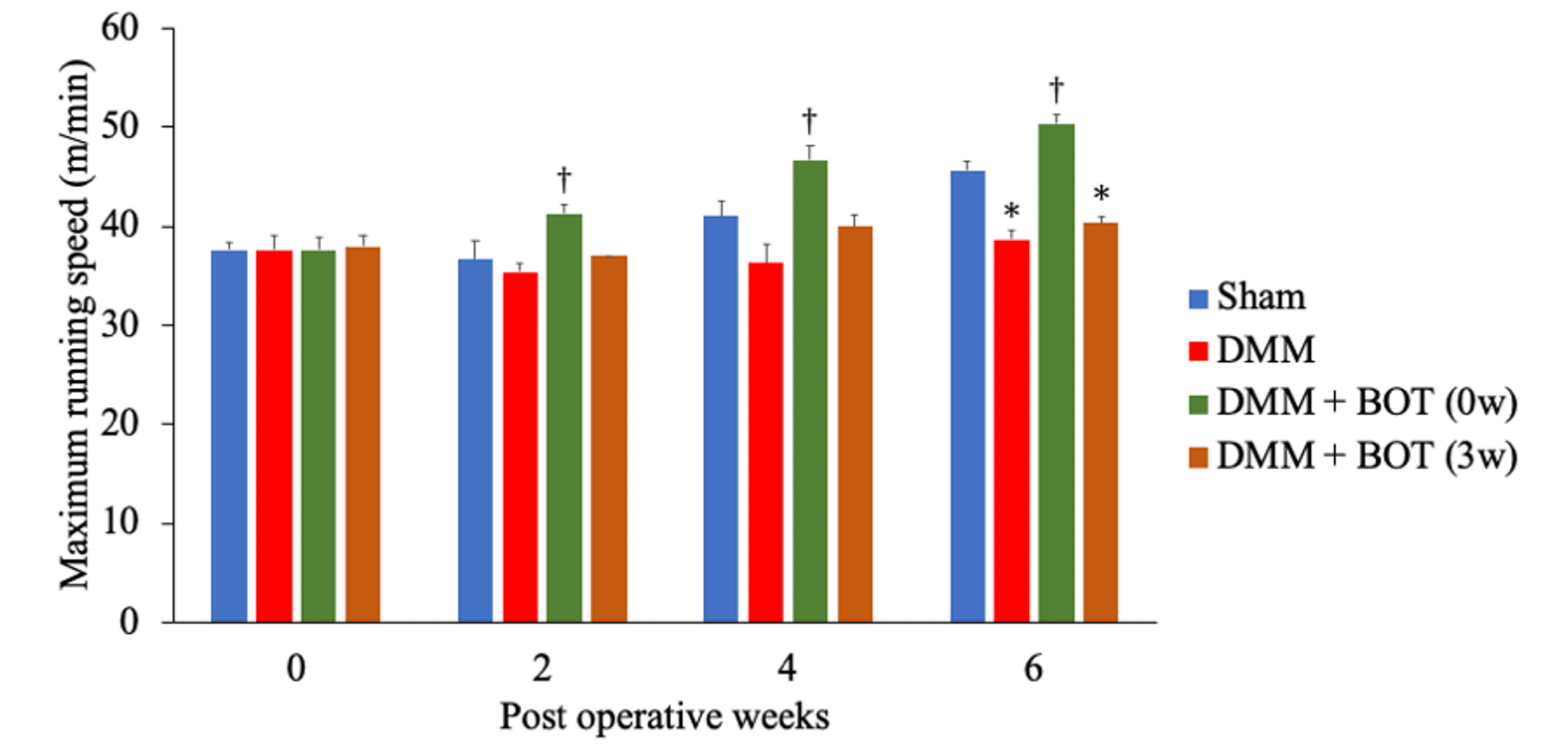
Treadmill running test Treadmill running tests were conducted during the 6-week experimental period following DMM surgery. The rats were made to run on a gradually accelerating treadmill, and their maximum running speed was obtained. No significant differences in exercise capacity were observed before DMM surgery. At 6 weeks post-surgery, the DMM and DMM + BOT (3w) groups had a significant decrease in exercise capacity compared with the Sham group. In contrast, the DMM + BOT (0w) group exhibited significantly higher exercise capacity compared to the DMM group. *P < 0.05 vs. the Sham group, ^†^*P* < 0.05 vs. the DMM group based on the Tukey test. BOT: boiogito, DMM: destabilization of the medial meniscus.

The maximum running speeds prior to DMM induction were 37.7 ± 0.7 m/min in the Sham group, 37.7 ± 1.3 m/min in the DMM group, 37.7 ± 1.2 m/min in the DMM + BOT (0w) group, and 38.0 ± 1.2 m/min in the DMM + BOT (3w) group; there were no significant differences among the groups. At 2 weeks post-DMM surgery, the maximum running speeds were 36.7 ± 1.9 m/min in the Sham group, 35.3 ± 0.9 m/min in the DMM group, 41.3 ± 0.9 m/min in the DMM + BOT (0w) group, and 37.0 ± 0.0 m/min in the DMM + BOT (3w) group; the DMM + BOT (0w) group had a significantly higher speed compared with the Sham group. At 4 weeks post-DMM surgery, the maximum running speeds were 41.0 ± 1.5 m/min in the Sham group, 36.3 ± 1.9 m/min in the DMM group, 46.7 ± 1.5 m/min in the DMM + BOT (0w) group, and 40.0 ± 1.2 m/min in the DMM + BOT (3w) group; the DMM + BOT (0w) group had a significantly higher speed compared with the DMM group. At 6 weeks post-DMM surgery, the maximum running speeds were 45.7 ± 0.9 m/min in the Sham group, 38.7 ± 0.9 m/min in the DMM group, 50.3 ± 0.9 m/min in the DMM + BOT (0w) group, and 40.3 ± 0.7 m/min in the DMM + BOT (3w) group; both the DMM and DMM + BOT (3w) groups had significantly lower speeds compared with the Sham group. Furthermore, the DMM + BOT (0w) group had significantly higher speeds from 2 weeks post-surgery onward compared with the DMM and DMM + BOT (3w) groups (Fig. 3).

**Figure 4:**
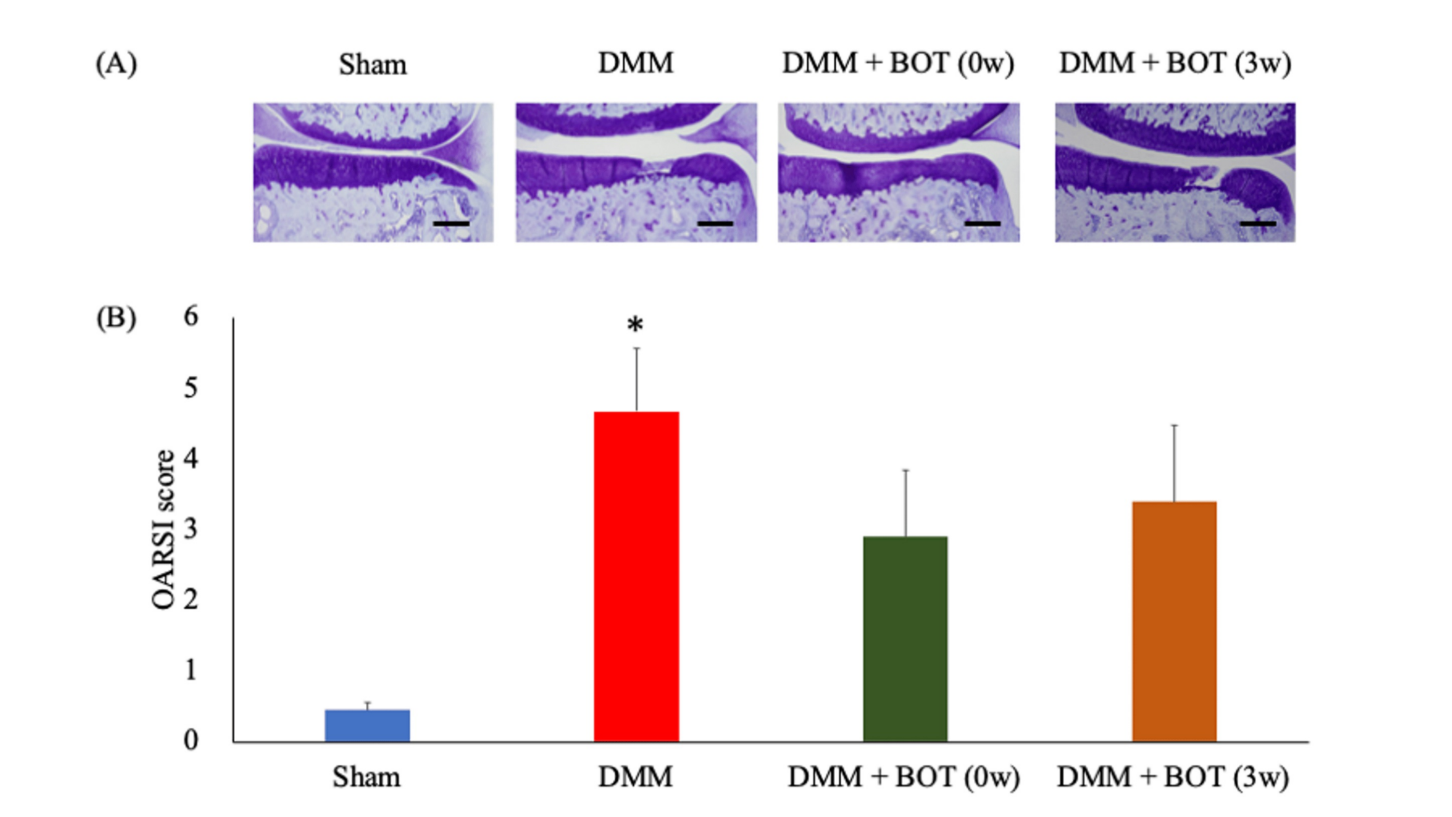
Histological analysis of the knee osteoarthritis score (A) Representative images of toluidine blue-stained right knee samples from each group. Magnification: ×40. Scale bars = 200 µm. (B) OARSI score. *P < 0.05 vs. the Sham group, ^†^*P* < 0.05 vs. the DMM group based on the Tukey test. BOT: boiogito, DMM: destabilization of the medial meniscus, OARSI: Osteoarthritis Research Society International.

Representative images of the results of toluidine blue staining for each group are shown in Fig. 4A. The scores were 0.4 ± 0.1 points in the Sham group, 4.7 ± 0.9 points in the DMM group, 2.9 ± 0.9 points in the DMM + BOT (0w) group, and 3.4 ± 1.1 points in the DMM + BOT (3w) group. The DMM group had significantly higher scores compared with the Sham group, while the DMM + BOT (0w) group had lower scores compared to the DMM and DMM + BOT (3w) groups (Fig. 4B), albeit without significance.

**Figure 5:**
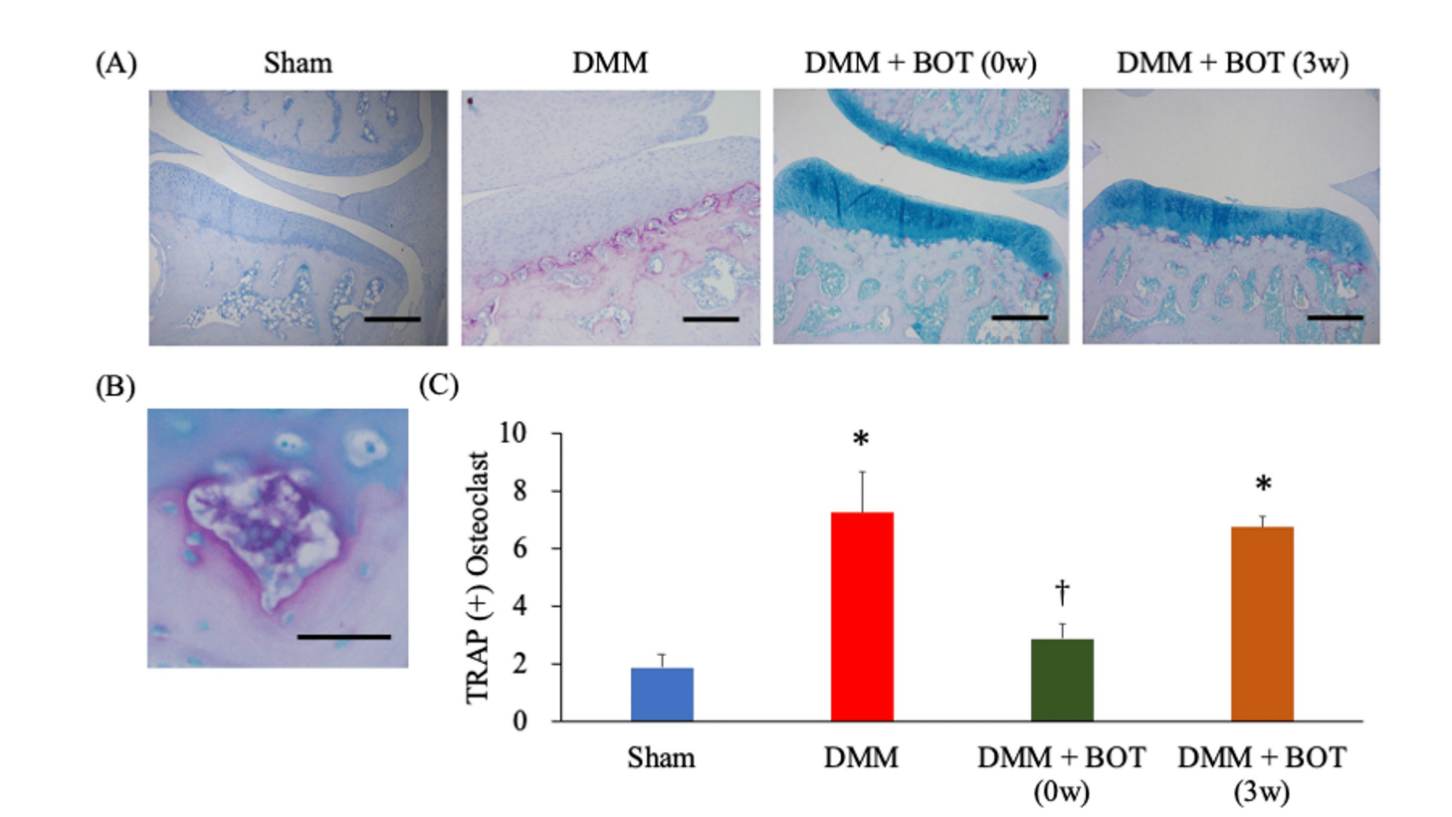
Histological analysis for quantifying TRAP-positive osteoclasts in subchondral bone (A) Representative image of TRAP staining of the right knee samples from each group. Magnification: ×40. Scale bars = 200 µm. (B) Multinucleated TRAP-positive osteoclasts. Magnification: ×400. Scale bars = 20 µm. (C) Number of TRAP-positive osteoclasts in the subchondral bone. *P < 0.05 vs. the Sham group, ^†^*P* < 0.05 vs. the DMM group based on the Tukey test. BOT: boiogito, DMM: destabilization of the medial meniscus, TRAP: Tartrate-resistant acid phosphatase.

Representative images of TRAP staining for each group are presented in Fig. 5A and Fig. 5B. The scores were 1.9 ± 0.4 in the Sham group, 7.2 ± 1.5 in the DMM group, 2.9 ± 0.5 in the DMM + BOT (0w) group, and 6.7 ± 0.4 in the DMM + BOT (3w) group. The DMM and DMM + BOT (3w) groups had significantly higher scores. Meanwhile, the DMM + BOT (0w) group had significantly lower scores compared with the DMM group (Fig. 5C).

**Figure 6:**
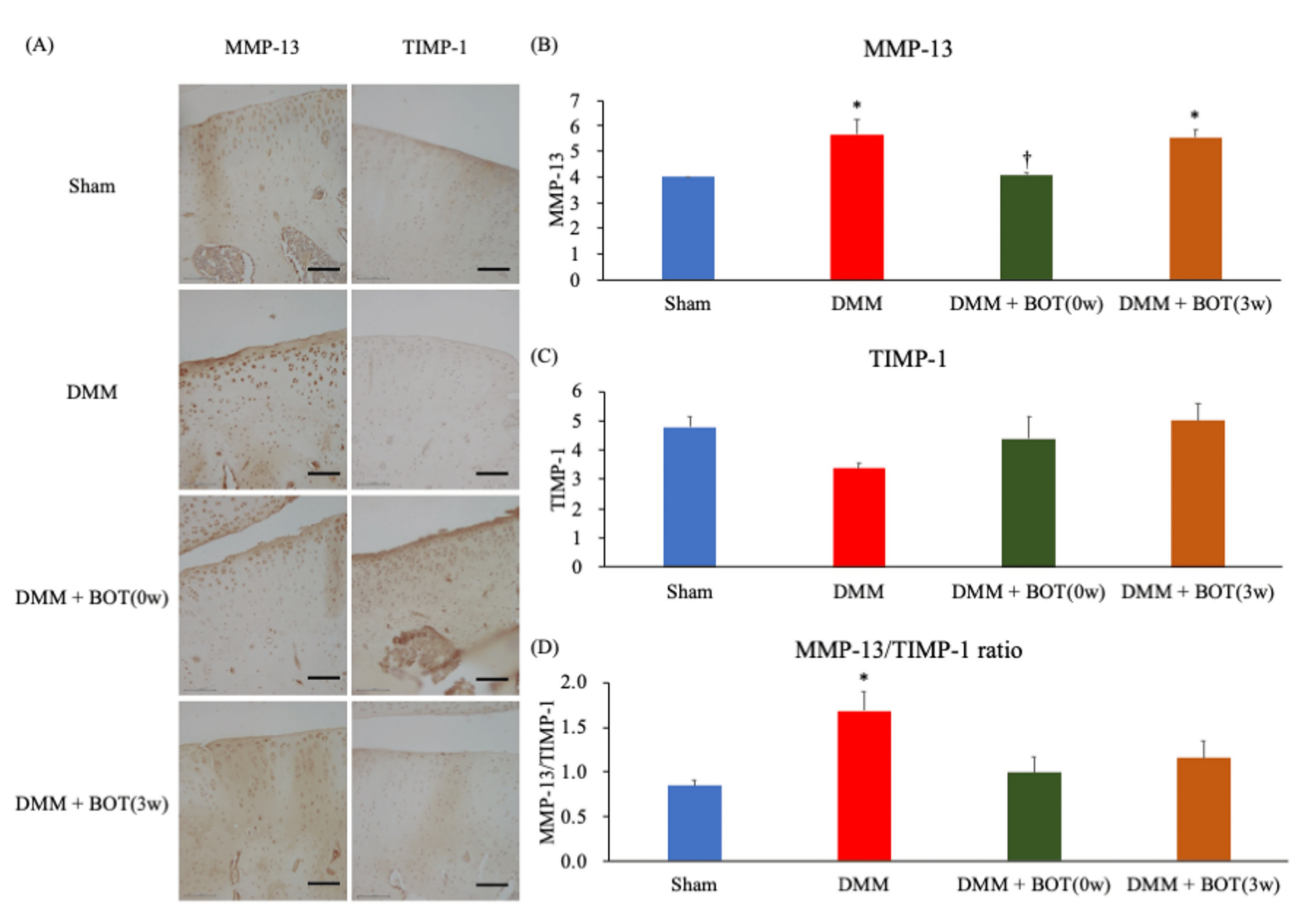
Immunohistochemical staining to evaluate the production and secretion of MMP-13 and TIMP-1 (A) Representative image of immunohistochemical staining for MMP-13 and TIMP-1 in right knee samples from each group. Magnification: ×200. Scale bars = 50 µm. (B) Score of the semiquantitative analysis for MMP-13. (C) Score of the semiquantitative analysis for TIMP-1. (D) Score of the MMP-13/TIMP-1 ratio. *P < 0.05 vs the Sham group, ^†^*P* < 0.05 vs. the DMM group based on the Tukey test. BOT: boiogito, DMM: destabilization of the medial meniscus, MMP-13: matrix metalloproteinase-13, TIMP-1: tissue inhibitors of matrix metalloproteinase-1.

Representative immunohistochemical staining images for MMP-13 and TIMP-1 are presented in Fig. 6A. The MMP-13 scores were 4.0 ± 0.0 points in the Sham group, 5.7 ± 0.6 points in the DMM group, 4.1 ± 0.1 points in the DMM + BOT (0w) group, and 5.6 ± 0.3 points in the DMM + BOT (3w) group. The DMM and DMM + BOT (3w) groups had significantly higher scores compared with the Sham group, whereas the DMM + BOT (0w) group had significantly lower scores compared with the DMM group (Fig. 6B). The TIMP-1 scores were 4.8 ± 0.4 points in the Sham group, 3.4 ± 0.2 points in the DMM group, 4.4 ± 0.7 points in the DMM + BOT (0w) group, and 5.0 ± 0.6 points in the DMM + BOT (3w) group. No significant differences were observed among the groups for TIMP-1 scores (Fig. 6C). Meanwhile, the MMP-13/TIMP-1 ratio was 0.8 ± 0.1 in the Sham group, 1.7 ± 0.2 in the DMM group, 1.0 ± 0.2 in the DMM + BOT (0w) group, and 1.2 ± 0.2 in the DMM + BOT (3w) group. Only the DMM group had a significantly higher ratio compared with the Sham group. Although the BOT-treated groups showed no significant differences compared with the DMM group, they also showed no significant differences compared with the Sham group (Fig. 6D).

## Discussion

The etiology of primary KOA is unclear. Meanwhile, approximately 13% of all KOA cases are classified as secondary KOA that results from trauma, infection, or other factors [17]. Studies have explored the progression of secondary KOA following knee trauma, particularly after ACL injury. KOA progression after ACL injury is faster compared with primary KOA, and the age at which surgery becomes necessary is approximately 10 years younger than that with primary KOA [18]. Recently, there has been growing attention on secondary KOA following meniscal injury, and it is recommended to proactively perform meniscal repair surgery on patients with meniscal tears [19].

The rationale is that the loss of the meniscus’ hoop function, which is important for stabilizing joint motion and distributing mechanical load, results in a disproportionate increase in joint surface stress, reaching 200% of normal levels. This excessive load disrupts the joint structural homeostasis [20]. Additionally, posterior root tears of the medial meniscus have gained attention as they are caused by damage to the ligament connecting the meniscus to the tibia. As people age, the medial meniscotibial ligament becomes fragile, rendering it vulnerable to rupture even under mild stress such as during sudden squatting. Medial meniscus posterior root tears (MMPRT) is a newly recognized pathology that was incidentally discovered through advanced imaging techniques, including magnetic resonance imaging [21]. It is highly likely that the onset and progression of KOA associated with MMPRT were encompassed within the broader category of primary KOA. 

In this study, an animal model replicating MMPRT was created to explore the effects of BOT on KOA progression. The findings showed that BOT administration in KOA model rats attenuated the deterioration of motor function related to disease progression and preserved the structural integrity of joint tissues. Additionally, BOT inhibited osteoclast proliferation in subchondral bone and mitigated the extent of cartilage degradation in articular cartilage. The differential impact of BOT on the suppression of KOA progression was notable as it depended on the timing of administration. Reductions in the number of osteoclasts in subchondral bone and lower semiquantitative MMP-13 scores were observed mostly in the DMM + BOT (0w) group. This indicates that administration of BOT immediately following DMM induction is more effective in attenuating degenerative changes in both subchondral bone and articular cartilage. Furthermore, early intervention with BOT following an injury is important to effectively attenuate the progression of secondary KOA. These results provide further insights into the potential clinical application of BOT in the treatment and management of KOA.

Several studies have revealed that pathological changes primarily affect the subchondral bone before the articular cartilage in the early stages of KOA progression. In the DMM model, subchondral bone turnover occurred earlier than 2 weeks [14], indicating that changes in subchondral bone may precede observable changes in articular cartilage during the early stages of KOA. Similarly, Iijima et al. [22] created a DMM rat model to explore the early KOA changes, revealing that the loss of meniscal function resulted in cartilage degeneration and subchondral bone defects, particularly in the middle region of the joint. Additionally, these defects were characterized by fibrous tissue that expressed MMP-13 and vascular endothelial growth factor, indicating early pathological changes in both cartilage and subchondral bone. These studies emphasize that the progression of early KOA involves pathological changes in subchondral bone that may precede or coincide with cartilage degeneration. 

MMP-13 is a key mediator of articular cartilage degeneration in osteoarthritis. MMP-13 is a matrix metalloproteinase that plays an important role in cartilage degradation due to its unique ability to cleave type-2 collagen [23]. This property makes MMP-13 a key contributor to the development and progression of KOA and a potential target for novel inhibitors to halt disease progression. In contrast, TIMP-1 is an endogenous inhibitor of metalloproteinases that strongly inhibits MMPs and specific members of the disintegrin and metalloproteinase with thrombospondin motifs family. TIMP-1 plays an important role in maintenance and remodeling of the extracellular matrix [24]. Using an ACL injury rat model, Onitsuka et al. revealed that there was a significant increase in the MMP-13/TIMP-1 ratio in the disease group that developed KOA [15]. An increase in the MMP-13/TIMP-1 ratio represents an exacerbation of articular cartilage degradation; conversely, a decrease in this ratio would indicate a protective effect. Our findings indicate that oral BOT administration tended to reduce the MMP-13/TIMP-1 ratio, suggesting a potential protective effect on articular cartilage. 

BOT is an herbal medicinal formula that originated from China, and it has been used since approximately the 3rd century. Clinically, BOT alleviates symptoms such as knee joint effusion and pain, particularly in cases of lower limb edema. In patients with KOA, BOT administration improves mobility, including the ability to ascend stairs [25]. In a study using synoviocytes isolated from knee joints, BOT administration reportedly suppresses the secretion of periostin, which is involved in the progression of KOA [26]. 

BOT is composed of Sinomenium stem, Astragalus root, *Atractylodes lancea* rhizome, Jujube, Glycyrrhiza, and ginger; Sinomenium and Astragalus play important roles as key ingredients. Using the compound sinomenine derived from *Sinomenium acutum*, Wu et al. revealed that Sinomenine inhibits the inflammatory response in mouse chondrocytes via the Nrf2/HO-1 and nuclear factor-kappa B (NF-κB) signaling pathways [27]. Additionally, Sinomenine downregulated the production of cartilage matrix-degrading enzymes and suppressed cartilage destruction. These results indicate that Sinomenine may potentially attenuate the progression of KOA. Furthermore, Astragalus polysaccharide, which is a component of Astragalus, inhibits osteoclast formation and differentiation in vitro. Astragalus also downregulates the receptor activator of NF-κB ligand-induced osteoclastogenesis and reduces the expression of osteoclast marker genes such as NFATC1, TRAP, c-FOS, and cathepsin K [28]. The therapeutic effects of BOT are attributed to the combined anti-inflammatory and analgesic effects of its various constituent herbs. As KOA is characterized by the progressive involvement of several tissues, including the subchondral bone, articular cartilage, and synovium, the various mechanisms of action of BOT align with the pathophysiology of KOA and provide a rational basis for its use.

This study has several limitations. First, the sample size was relatively small, which may limit the generalizability of the findings. Second, while BOT mitigates KOA progression, the specific molecular mechanisms underlying this effect remain unclear. Third, the timing and duration of BOT administration were limited to specific intervals, leaving its long-term efficacy and potential benefits of delayed treatment unknown. Finally, the reliance on a single animal model may not fully capture the complexity of KOA in humans, necessitating further validation in diverse models and clinical studies.

## Conclusions

The present study demonstrated that early administration of Boiogito (BOT) mitigates the progression of knee osteoarthritis (KOA) in a rat model by preserving motor function and reducing pathological changes in both the subchondral bone and articular cartilage. BOT notably suppressed osteoclast proliferation and reduced cartilage degradation, as evidenced by lower TRAP-positive osteoclast and MMP-13/TIMP-1 ratios in the DMM + BOT (0w) group. These findings underscore the importance of initiating treatment soon after meniscal injury to achieve optimal therapeutic outcomes, highlighting the potential of BOT as an effective intervention for early-stage KOA.

The multifaceted anti-inflammatory and cartilage-protective effects of BOT align with the complex pathophysiology of KOA, suggesting a promising clinical application for this traditional Japanese medicine. These findings provide a foundation for integrating BOT into therapeutic strategies aimed at inhibiting the progression of KOA and improving patients' quality of life.
